# Supplementary material for: Crossover of the dimensions of work-family and family-work conflict in couples: Protocol for a qualitative study
Source: PLoS One. 2023 Sep 28;18(9):e0290216. doi: 10.1371/journal.pone.0290216 (PMC10538721; doi:10.1371/journal.pone.0290216)
Supplement: S1 File — (DOCX) [file pone.0290216.s001.docx]

**Supporting Information 1. Interview guide.**

*Note: At this point, interviewees will have already been familiarized with interview goals, filled out demographic survey and signed informed consent before meeting the interviewer.*

Hello, my name is XY, and I will conduct this interview. Thank you for joining me today. As a reminder, let me say that participation in this interview is voluntary, and you can resign from the conversation at any time. You can also ask for any information that you provide to be deleted. We will only record the audio of this conversation and the data that we collect, that is, your responses, will be anonymous. Nevertheless, transcriptions will be stored on a computer and will be password-protected. The consent form that you signed earlier will be stored separately from the transcriptions and is also password protected.

**Are the terms of the interview understandable to you?**

▢Yes

▢No (if not - we explain doubts)

**Do you maintain your consent to participate in the interview?**

▢Yes

▢No (if not - end the interview)

| **Domain** | **Sample Questions** |
| --- | --- |
| Warm up | Tell me about your work, please. |
| RQ1a: What dimensions of WFC and FWC do partners identify in themselves? | When you hear “work and family” what is the first thing that comes to mind?  When balancing work and family, do you face any challenges or difficulties? Can you please tell me about those?  *Note: If the dimensions do not come up naturally, we will ask about them specifically:*  Tell me about the role that time plays in this conflict.  Tell me about the role that fatigue plays in this conflict.  Do you sometimes feel the need to be a different person at work and at home? Tell me what role this plays in the conflict between work and family.  *Note: If all the responses focus on WFC, we will specifically ask about FWC:*  So far, we talked about how work affects your family life. Let’s now look at the other direction: tell me about a situation or situations when your non-work-related life made it harder for you to meet demands at work? What happened then?  *Note: If the dimensions do not come up naturally, we will ask about them specifically (see questions above)* |
| RQ1b: What dimensions of WFC and FWC do partners identify in their partners? | Now let’s talk about your partner’s situation. Tell me, how the balancing of work and family looks in their case.  How do you recognize that your partner experiences conflict between work and family?  What makes it particularly hard for your partner to balance work and family?  *Note: If the dimensions do not come up naturally, we will ask follow-up questions similar to those in RQ1a*  *Note: If all the responses focus on WFC, we will specifically ask about FWC:*  So far, we talked about how your partner’s work affects their family life. Let’s now look at the other direction: tell me about a situation or situations when their non-work-related life made it harder for them to meet demands at work? What happened then?  *Note: If the dimensions do not come up naturally, we will ask about them specifically (see questions above)* |
| RQ2: How do different dimensions of WFC and FWC cross over between partners? | Earlier we talked about how your partner experiences situations when it is difficult for them to meet demands at home due to work obligations. Can you tell me how this work to family conflict that your partner experiences affects you?  Do you react differently depending on whether your partner’s conflict stems from them: not being able to catch up due to time constraints, being too tired at home, or acting at home as if they were at work?  And now, just as earlier, let’s look in the opposite direction, when your partner has difficulties with meeting demands at work because of what’s going on at home. What happens then? How does this family to work conflict that your partner experiences affect you?  Do you react differently depending on whether your partner’s conflict stems from problems with making time, lacking energy or the need to act differently at work than at home? |
| RQ3: What coping strategies with WFC and FCW on an individual and dyadic level do partners identify? | What do you do yourself and what do you do together with your partner to manage the difficulties in balancing work and family? |
| RQ4: What potential barriers and facilitating factors in participating in an internet intervention designed to reduce WFC and FWC do partners recognize? | In the future, we plan to design a psychological program to help couples in managing conflict between work and life. To make it widely available and ensure flexibility in usage we plan to deliver it online, probably on a dedicated, interactive website. On this website, there will be a number of tasks to be completed either alone or with a partner. Because it will be online, it will be possible to use it from any location and at a time that works for people who use it. Imagine that you are offered to use such a program together with your partner.   - Could you tell me what would make it easier for you and your partner to join such a program and keep using it for a few weeks? - And what could prevent either of you from joining such a program or continuing to use it for a few weeks? |
| Ending | Is there anything that I didn’t ask about and you feel that is important in the realm of the conflict between work and family? |

Thank you for your participation and for sharing your experiences. Should you have any follow-up questions, you can contact the agency that is responsible for recruitment or our research team directly.
